# Delivering and participating in a psycho-educational intervention for family caregivers during palliative home care: a qualitative study from the perspectives of health professionals and family caregivers

**DOI:** 10.1186/s12904-015-0015-1

**Published:** 2015-04-24

**Authors:** Maja Holm, Ida Carlander, Carl-Johan Fürst, Yvonne Wengström, Kristofer Årestedt, Joakim Öhlen, Anette Henriksson

**Affiliations:** Department of Neurobiology, Care Sciences and Society, Division of Nursing, Karolinska Institutet, Stockholm, Sweden; Palliative Research Centre, Ersta Sköndal University College, Stockholm, Sweden; Department of Learning, Informatics, Management and Ethics, Karolinska Institutet, Stockholm, Sweden; Department of Clinical Science, Lund University and the Institute for Palliative Care at Lund University and Region Skåne, Lund, Sweden; School of Health and Medical Sciences, Örebro University, Örebro, Sweden; Department of Medical Health Sciences, Linköping University, Linköping, Sweden; Center for Collaborative Palliative Care, Linnaeus University, Kalmar, Sweden; Centre for Person-Centred Care and Institute of Health and Care Sciences, Sahlgrenska Academy, University of Gothenburg, Gothenburg, Sweden; Department of Health Care Sciences & Palliative Research Centre, Ersta Sköndal University College, Stockholm, Sweden; Capio Geriatrics, Palliative Care Unit, Dalen Hospital, Stockholm, Sweden

**Keywords:** Family caregivers, Health professionals, Home care, Psycho-educational intervention, Palliative care, Support

## Abstract

**Background:**

Family caregivers in palliative care have a need for knowledge and support from health professionals, resulting in the need for educational and supportive interventions. However, research has mainly focused on the experiences of family caregivers taking part in interventions. To gain an increased understanding of complex interventions, it is necessary to integrate the perspectives of health professionals and family caregivers. Hence, the aim of this study is to explore the perspectives of health professionals and family caregivers of delivering and participating in a psycho-educational intervention in palliative home care.

**Methods:**

A psycho-educational intervention was designed for family caregivers based on a theoretical framework describing family caregiver’s need for knowing, being and doing. The intervention was delivered over three sessions, each of which included a presentation by healthcare professionals from an intervention manual. An interpretive descriptive design was chosen and data were collected through focus group discussions with health professionals and individual interviews with family caregivers. Data were analysed using framework analysis.

**Results:**

From the perspectives of both health professionals and family caregivers, the delivering and participating in the intervention was a positive experience. Although the content was not always adjusted to the family caregivers’ individual situation, it was perceived as valuable. Consistently, the intervention was regarded as something that could make family caregivers better prepared for caregiving. Health professionals found that the work with the intervention demanded time and engagement from them and that the manual needed to be adjusted to suit group characteristics, but the experience of delivering the intervention was still something that gave them satisfaction and contributed to them finding insights into their work.

**Conclusions:**

The theoretical framework used in this study seems appropriate to use for the design of interventions to support family caregivers. In the perspectives of health professionals and family caregivers, the psycho-educational intervention had important benefits and there was congruence between the two groups in that it provided reward and support. In order for health professionals to carry out psycho-educational interventions, they may be in need of support and supervision as well as securing appropriate time and resources in their everyday work.

## Background

As health care systems in many countries are moving towards outpatient care, family caregivers have been given increased responsibility for patients with incurable illnesses [1]. Reviews of the literature have shown that family caregivers in palliative home care have a need for better communication with health professionals and that their needs for psychosocial support and information are often unmet [2,3]. In the context of palliative care, a family caregiver could be defined as any relative, friend, or partner who has a significant relationship with and provides various forms of support to a person with incurable illness [4]. Family caregivers may experience a considerable burden when caring for a person with complex and serious conditions, which could affect their health and wellbeing negatively [5-7]. Both family caregivers and health professionals have reflected upon what they feel is a lack of confidence and limited knowledge in family caregivers [8]. A caregiver has a greater chance of adapting to the situation if he or she feels capable and has resources at their disposal. Such resources include feelings of preparedness, competence, having adequate information, and focusing on positive aspects of the situation [9]. Qualitative findings demonstrate that family caregivers need to be recognized and acknowledged by health professionals, as they may find it difficult to ask professionals for help and information in their situation as caregivers [10,11].

Home-based family caregivers have a documented need for knowledge concerning symptoms and symptom relief, comfort, nutrition, personal care and technical equipment. The significance of effective communication and information-sharing between patient, caregiver and health professionals has also been emphasized. Research has consistently highlighted that family caregivers lack practical support, often related to inadequate information. Such deficits could lead to family caregivers adopting a ‘trial and error’ approach to palliative caregiving. Enhanced possibilities for communication and discussions with health professionals represent a potentially effective method of increasing caregivers’ confidence in their ability to undertake practical aspects of home-based care. Evidence suggests that health professionals may better assist home-based family caregivers by providing them with information and skills-training. This may necessitate the development of new educational interventions aimed at supporting family caregivers [12,13].

There is often a lack of theoretical or conceptual frameworks guiding the development and delivery of supportive interventions for family caregivers [7]. A theoretical framework has been developed by Andershed and Ternestedt, regarding the involvement of family caregivers in palliative care, focusing on their need for knowing, being and doing. Knowing is both a part of the family caregiver’s involvement and a prerequisite for involvement in the form of being and doing; thus crucial in this context. Family caregivers try to increase their understanding of the patient’s condition by actively seeking knowledge of the patient’s symptoms, diagnosis, prognosis and so forth. Being concerns the emotional aspect of being a family caregiver, such as spending time with the patient and sharing his or her world. An example could be to take time away from one’s own work and to share emotions of grief and love. Doing has a more practical aspect and involves the family caregiver doing things for the patient that he or she would do for themselves if they were healthy, such as helping the patient with hygiene or medication or keeping in contact with friends and family [14,15].

It has been suggested that family caregivers need education and support for their own sake, to cope with their own situation with concerns such as problem-solving strategies, how to care for their family members, how to maintain their own health and how to deal with bereavement. In order to meet family caregivers’ crucial need for knowing and making sense of palliative care-related issues and information, they often depend on other people, such as health professionals [16].

Based on a review of intervention studies aimed at family caregivers in palliative care, different intervention models to provide support have been reported, including; individual interventions such as psychological support, palliative care/hospice interventions, information and training interventions, respite interventions, and physical interventions. The most common form was group interventions of different sorts [17]. An important advantage of group interventions is their relatively low financial costs and workload for health professionals supporting many family caregivers [18]. It has been pointed out that, considering their often limited time, group interventions directed at family caregivers should be brief and aim to increase their preparedness for their role, and provide information and emotional support (psycho-educational intervention) [19]. The use of supportive multi-disciplinary health professionals has been found to be a key strength in successful group interventions, but research has also shown that another important factor is the support and comfort exchanged between the family caregivers participating in the groups [20-22].

Group interventions for family caregivers in palliative care could be considered complex to evaluate because they include several components [23]. Previous research has demonstrated good effects on outcomes such as preparedness and competence for caregiving, but also that they have been experienced as meaningful, contributing to feelings of togetherness and safety in family caregivers [20,22,24,25]. Health professionals play an important role in family caregivers’ experience of participating in interventions [20,22] and it could be assumed that a successful intervention needs to be appealing to both health professionals and family caregivers. However, the authors have not found any studies focusing on both the perspectives of delivering and participating in a group intervention in the context of palliative care. Such studies could contribute to further development of complex interventions. Thus, the aim is to explore the experiences of delivering and participating in a psycho-educational intervention from the perspectives of health professionals and family caregivers in specialized palliative home care.

## Methods

### Design

The study had a qualitative approach and interpretive description was chosen. This design proposes that human experiences are socially constructed and related to social context. The goal is to achieve a coherent conceptual description of the clinical phenomenon the research is focused on and it is considered important to take personal and disciplinary fore-structures into account [26]. Therefore critical reflections in the research group were used as significant means to enhance study credibility throughout the process. These reflections especially included explication of knowledge fore-structure in relation to the study focus. Firstly, the theoretical underpinnings as described in the background; secondly, the researchers’ disciplinary backgrounds from nursing and clinical medicine implied an epistemological interest of contributing to practice knowledge and thirdly, the researchers in the team have various and complementary expertise as related to family caregiver support which was used for reflecting upon in what ways the analysis might mirror their conceptual fore-structures. Ethical approval for the study was obtained from The Regional Ethical Review Board in Stockholm (2012/4:3, 2012/377-31/4).

### Intervention development

A psycho-educational intervention, aiming to increase preparedness for caregiving in family caregivers, to support their wellbeing, and decrease negative consequences associated with caregiving, was developed in steps [20,25]. The intervention is based on the theoretical framework of Andershed and Ternestedt [15] relating to the concepts of knowing, being, and doing. The framework was used to construct an intervention manual, tailored to meet family caregivers’ need for knowing and doing caregiving as well as being with their sick family member. The manual consists of a compendium of evidence-based knowledge on different topics for health professionals to present and discuss in interaction with family caregivers. The manual was developed in collaboration between researchers and a reference group of health professionals during a one-day workshop covering the theoretical framework of the intervention and how it should be conducted. In addition, several meetings between researchers and health professionals took place pre-, during and post-intervention delivery. Health professionals were given an introduction to, and a chance to become familiar with, the manual, ask questions to the researchers and prepare for their delivery of the intervention. It was stated that although each of the topics of the manual should be covered, health professionals could allow for flexibility depending on the group’s characteristics and needs. For example, if the group had a particular interest in symptom relief, their thoughts and questions on this topic could be discussed. This strategy was considered to be in line with the psycho-educational approach of the intervention, focusing on both its educative and supportive aspects. Meetings were also arranged to give health professionals from the various settings a chance to share their thoughts and discuss the manual in order to ensure consistency across the intervention. They were also in regular contact and had discussions with the researchers in case they were in need of support or had questions regarding the process.

The intervention consisted of three sessions and the topics were presented by a member of the professional care team (physician, nurse, and social worker or priest). Each session was planned to last 2 hours. A nurse acted as group leader and participated in all meetings. The manual was used as a structure and support for the three sessions with different topics based on family caregivers’ knowing, being and doing. The manual was designed to involve all three concepts throughout the intervention, focusing on educational, practical and emotional topics related to family caregiving. Knowing represents the educational topics in the sessions; examples include: palliative diagnoses, trajectories and symptom relief at the physician session; hygiene and nutrition problems at the end of life, such as difficulties to eat, at the nurse’s session; and what grief reactions a family caregiver can expect at the social worker session. Knowing is also a precondition for the practical and emotional concepts through the intervention. Through knowing the family caregivers are helped to manage the practical caregiving (doing) and relate to their own feelings (being). A concrete example from the manual could be the nurse’s session regarding various eating difficulties at the end of life. Family caregivers are also given a chance to know how they can prepare food that is easier for the patient to eat; they have a chance to talk about their feelings and relate them to the experiences of other caregivers. The psycho-educational intervention provides family caregivers with an approach to support their knowledge-seeking (knowing) which could make caregivers better prepared for both emotional (being) and practical (doing) aspects of caregiving (Table 1).

**Table 1:** **Intervention content, topics, structure of the intervention**

	**Meeting 1 group leader (nurse) + physician**	**Meeting 2 group leader (nurse)**	**Meeting 3 group leader (nurse) + social worker, or priest**
**Topic for the meeting**	Palliative care and symptom management.	Daily life and practical nursing care	Emotional reactions and grief
**The participants arrive**	The group leader welcomes and participants are offered coffee/tea and snacks.		
**Topic for the day (60-90 min)**	A professional presents the topic of the day. Participants are invited to engage in a dialogue.		
**Reflection (20-30 min)**	Participants are invited to reflect upon the topic of the day		
**Conclusion and relaxation practice (10 min)**	Conclusion and a short relaxation practice guided by the group leader.		

### Settings

The intervention was delivered by health professionals at 10 specialized palliative home care settings in a metropolitan area in Sweden. The health professionals provided palliative care for patients with complex needs and limited survival expectancy; regardless of diagnosis. A majority of the patients were affected by malignant disease with a metastatic progress, but the settings also provided care for patients with other conditions, such as chronic obstructive pulmonary disease, cardiovascular diseases, and neurological diseases. The needs of the patients included symptom management, emotional and spiritual support, and assistance with personal nursing care. All settings delivered 24-hours-a-day services and were staffed by multi-professional teams including physicians, nurses, social workers, priests and occupational and physical therapists. The settings employed between 30 and 90 professionals and had a capacity for 70 to 200 patients and also offered inpatient care (between 10 and 20 beds at each setting).

### Data collection

Between January 2013 and April 2014, each care setting successfully delivered the psycho-educational intervention 1-4 times. Family caregivers were approached and invited to the intervention by health professionals. They were given an invitation brochure and written information that the intervention was delivered as a research study; that participation was voluntary with possibilities to withdraw at any time and that the patient’s care would not be affected. Caregivers were invited according to the following inclusion criteria: being family caregiver to a person in specialized palliative home care, over the age of 18 and being able to read and understand the Swedish language. In all, 98 family caregivers participated in the intervention and a total of 21 intervention groups were conducted. The groups had an average number of 4 participants. Data for the present study were collected through focus groups with health professionals; and individual interviews with family caregivers, either face-to-face or by telephone. Written informed consent was obtained from all participants in the study.

#### Focus group discussions with health professionals

Health professionals (n = 40) who were involved in delivering the intervention at the 10 settings were invited to the research centre to take part in the focus group discussions. Altogether, 25 health professionals (24 women and 1 man) aged 30 to 63 years, accepted the invitation. The participants had between 3 and 20 years of experience of working in palliative care. They received written information about the study and its purpose with a request for them to participate. The largest group of participants consisted of nurses who had acted as group leaders for the intervention at their settings. The group leaders were mainly responsible for the inviting of family caregivers to the intervention and the communication with the study researchers. The two other groups of health professionals consisted of physicians and social workers or priests from the included settings (Table 2).

**Table 2:** **Sample of health professionals**

**Professional category**	**Focus group session 1**	**Focus group session 2**
**Nurse**	12	7
**Physician**	2	2
**Social worker/Priest**	5	1

Focus group discussions were carried out at two time points, three groups and two groups respectively, within a time period of six months in-between in order to possibly increase variation; experiences from the early phase of delivering the intervention as well as those from after having delivered several sessions. The researchers participated in the focus groups as moderators. Two researchers moderated the focus groups; one of them acted as facilitator and guided the health professionals, focusing on the professionals’ experiences of the intervention, its design and purpose, the invitation process, the manual, the actual delivery, reactions from family caregivers and other colleagues and its importance, while the other took notes and asked probing questions for clarification. The focus group discussions were audiotaped.

#### Individual interviews with family caregivers

To gain access to the perspectives of family caregivers, individual interviews with family caregivers were carried out after they had completed their participation in the intervention. Family caregivers were theoretically sampled based on their participation in the intervention groups. The focus was to obtain maximum variation [27]; hence we aimed to include caregivers from different care settings, of different sexes, ages and different relations to the patient in order to gain a spread of experiences and perspectives of their participation in the groups. Data was collected during two time periods, starting with participants from groups held during spring 2013 and continued with participants from intervention groups in spring 2014. In total, six palliative settings were represented. Family caregivers received written information about the study and its purpose with a request to participate in an interview. This request was then followed up by the first author with a telephone call. Of the 14 invited family caregivers, 13 accepted participation; 3 men and 10 women They were between 38 and 93 years old and were either spouses (n = 8) or children (n = 5) of the patient. The interviews were conducted by the first author; 6 face-to-face and 7 by telephone, using an interview guide with open-ended questions, concerning the family caregivers’ experience of participating in the intervention, the intervention topics and its perceived strengths and weaknesses. The interviews were all audiotaped.

### Analysis

The two datasets from the health professionals and family caregivers were transcribed verbatim and analysed separately, applying the framework analysis approach (FA) [28]. The FA was used similarly for both the datasets, as a pragmatic approach for the management of the two qualitative datasets. Thus, it offered a structured process for the analysis in line with the principles of interpretive descriptive design whilst also allowing for the flexibility associated with qualitative inquiry.

The FA began with the authors reading through the transcripts of the data several times to become familiar with them. Emerging ideas, represented by themes, were noted, discussed and added in a developing framework, based on the research question of finding out the perspectives of those who delivered and participated in the psycho-educational intervention. Excerpts which were linked to the same theme were grouped together. The material was reduced to brief summaries of what was said by participants, creating an analytical framework. The summaries and themes were then compared to the original data to see if any further changes were required. The results from the analysis of the two datasets were then merged into a common result with a focus on similarities and variations in the experiences, both within and between the two groups.

## Results

### Inviting participants and being invited to participate in the intervention

The health professionals involved in the delivery of the intervention emphasized that they had put much effort in inviting appropriate caregivers for participation. They spent a considerable amount of time with the rest of the care team going through patient records, making sure the patient was diagnosed to be in a palliative phase of their illness and had received information about the palliative orientation of care from the physicians. There was a sense of uncertainty among the health professionals when targeting patients and family caregivers who might be hurt by the intervention content, as it included topics such as crisis reactions, dying and death. Some of the health professionals reflected upon whether they might have been too cautious in their invitation process. The nurses who acted as group leaders described the procedure of inviting as more difficult when it came to the family caregivers of patients with non-malignant diseases.

*“We had these multi-morbid patients that we were going to invite … they are palliative as well … it’s COPD or heart failure. They have a palliative diagnosis … We actually got this woman with a husband with a heart disease and she was really satisfied and thought everything was great. But it’s really quite difficult.”*

*“We also had many of those. We only choose those in the late phase of heart failure and COPD since they can live on for so many years so we made that choice with the nurses from that group.”*

*“We have three teams, two with a cancer profile and one with a general medical profile and we didn’t include them, we only concentrated on the cancer patients. It was easier to handle it this way or it would have been too much.”*

Whilst all the family caregivers recognized the general relevance of the intervention, there were also family caregivers who questioned whether they were suited for the intervention. They viewed the intervention as targeting family caregivers taking an active part in the practical and medical care of a patient, which they were not all doing. There were also reflections on whether their family members were as sick as the intervention required. However, family caregivers also pointed out that the patient’s condition would likely deteriorate and that the intervention could prepare them in future for a more active role as caregivers. Others held the opposite opinion; that the intervention was aimed at a basic level of care and because they had long been involved in advanced caregiving, the topics did not present much that was new to them. However, the dilemma of balancing different needs among family caregivers was voiced by a man caring for his wife as he stated that the intervention could not be designed to fit everyone’s individual situation, but rather that it should be focused on a wider population of family caregivers.

*“I imagine that I am very atypical in this case so you can’t do something that would fit for just me. Then I think it would be too narrow for others, who are not as actively involved in caregiving as I am.”*

There were also expressions of satisfaction from the family caregivers in that the groups were heterogeneous and included family caregivers of different backgrounds, with patients of different diagnoses and at different stages of the palliative trajectory. The group differences allowed them to exchange their various experiences and learn from each other. Other family caregivers had the opposite wish; that the groups should have been more homogenous with participants in more or less the same situation. They felt that this would have been more beneficial to them.

### The intervention topics as a support and framework for relevant discussions

Before delivery of the intervention, the health professionals needed time to read the intervention manual through in detail and to learn how to use it. This was considered to be time-consuming and was often done outside working hours. Opinions of the manual were that it was perceived as adequate and relevant for the aim of the intervention. However, it was also viewed as comprehensive and detailed, which, for some individuals, was associated with feelings of pressure and burden.

Even though they felt obliged to cover all the topics of the manual, health professionals described that they had tried to deliver the manual content in their own way, with their own words. Because all the participating health professionals were familiar with and experienced in palliative care, the manual was considered to be a supportive tool to pinpoint central aspects in the topics included. Sometimes they left parts of the manual out and sometimes they focused more on specific areas due to questions and discussions among group participants. All the health professionals considered it essential to be flexible in the use of the manual and to adjust it to the group participants with regards to age, relationship to the ill person, individual approaches to being in the group, for example being talkative, silent or emotional, and the broad variety of backgrounds and experiences. One physician stated that she found the experience of deciding where to put the focus very stimulating.

*“It was exciting to step in there … to quickly understand what kind of audience I had … so it was everything from a … well, perhaps she wasn’t a teenager, but clearly a younger girl to gentlemen in their middle age … and everything in between …”*

Among family caregivers, the topics of the manual were considered to be interesting and useful to family caregivers in general. The content of the manual was described as something that could prepare them for the deterioration of the patient, a matter that was viewed as important. One participant found that she could draw strength from the topics even if they were not always relevant to her situation.

*“Yes, I thought it was great since they divided the things in a good way. When the doctor was there, she spoke a lot about palliative care and of course I could feel a little bit left out since we have not reached that stage or whatever you should call it. But my mum passed away a year and a half ago so then I experienced all this with her. She lived in a nursing home, but we spent a lot of time with her and we could follow this process. So in that way I felt like home. And I know what is going to happen. My dad is 94 years with cancer in three places so you know that, out of the blue, you might be in that situation once again.”*

The manual covered difficult topics such as death and grief and the participants appreciated the opportunity to discuss and reflect on these matters even if they were difficult and emotional. The intervention sessions were considered to be a forum for them as family caregivers and the topics presented offered a framework for the sessions that allowed them to open up and engage with the others in the group.

### Finding rewards and frustrations in delivering and participating in the intervention

Preparing for the intervention delivery demanded personal engagement and responsibility from the health professionals, including practical arrangements and meetings with the team to make sure invitations were given out. Some were given extra time for the intervention delivery, while others received less acknowledgment from their employers. Some professionals were experienced in providing group interventions, while most of the professionals had no previous experience in delivering supportive and/or educational interventions, which contributed to feelings of tension. Despite the comprehensive pre-intervention work, all of the health professionals involved in the intervention expressed feelings of great satisfaction from delivering the intervention sessions and they talked about feeling rewarded, both professionally and personally. The meetings made the work of the preparations worthwhile.

*“It’s such a thrill. It’s just so amazing, both the discussions … it’s such a pleasure. The patients talk about it, the family caregivers talk about it. You could meet someone a long time after and they say “Hello, we met at the family group!” and they ask a lot of questions. I mean … the professional role … it’s a niche that is really powerful to be inside … it’s just such a cool thing.”*

“*Personally I think it just feels amazing, you feel strengthened and these caregivers you had in the group, you meet them with the patient and you are being told by your colleagues, they have been so happy, they thought everything was great … and they got so much back from it and I had not thought about that. I had not realized it.”*

The family caregivers gave many examples of feeling elevated by the intervention groups. They were able to exchange experiences and share suggestions of what could be done to manage and prepare for difficult situations with each other as well as with the health professionals. There were often emotional moments, both crying and laughter occurred in open discussions about sensitive matters such as the upcoming death of their ill family member. The professionals witnessed several situations when a participant was consoled and supported by the group. A daughter caring for her father believed the intervention gave a sense of togetherness and the atmosphere of the group was described as warm and tolerant of sharing experiences in confidentiality.

*“Something I thought presented itself very clearly is that you are not alone in feeling bad when you are not going to visit your dad. Those conversations were a relief, they gave confirmation. No, I am not selfish just because I want to do my own things as well. Perhaps that was the greatest reward.”*

Different opinions were expressed regarding the number of intervention sessions. Some felt that three was a suitable number, while others expressed a wish to have more sessions in the intervention. In some cases, family caregivers felt that relations had begun to be established in the groups, but that three sessions were not enough to develop them fully. They would also have liked more sessions to talk more freely without the intervention structure. However, family caregivers also pointed out that more sessions would have meant more drop-outs as patients would eventually die. Several health professionals also mentioned that they would have liked more sessions with their groups. In some cases, the family caregivers had decided to arrange their own meetings after the intervention was concluded or to continue having contact through e-mail.

### Focusing on family caregivers and feeling acknowledged by health professionals

The professionals emphasized that thanks to delivering the psycho-educational intervention the needs of family caregivers became something that was discussed within the teams and became a part of the daily work at each of the study settings. The professionals talked about the intervention as a means to build competence in family caregiver support and thereby profile their unit in this area. Meeting the family caregivers in groups, listening to questions and stories of their situation made the professionals even more sensitive to caregiver needs, both within the intervention and when meeting families during regular home visits. They found they had gained a greater insight, somewhat to their own surprise, as they considered themselves skilled experts in palliative care with long experience.

*“To me it was like a … light being turned on. I told my superior when I met her that I hadn’t thought about that … Like, I have been working for all these years but it had not occurred to me that they are going in and out of these processes all the time for several times … actually.”*

The family caregivers argued that being asked to participate in the intervention made them feel recognized by the health professionals. They felt that they received an acknowledgement that family caregivers have an important role and that they face a great responsibility. It also felt good to be able to do something on their own in their often demanding everyday lives as illustrated by a participant caring for her father.

*“But I think it is a really great thing and I think many family caregivers would need this kind of support group when you end up in this kind of situation, since your life is really changed, sometimes you sort of feel like your life isn’t your own. Now there is another human being that you suddenly have to be there for and help that you had not expected. Suddenly you need to give up a lot of your own things and try to reschedule your time.”*

Some of the professionals commented that the family caregivers in the intervention groups talked about being better prepared for their own situation, by listening to information and experiences described by others in a similar situation. Family caregivers also mentioned that the sessions included a closer contact with the palliative team and the different professionals. The intervention gave them an opportunity to ask questions and find out more about what the palliative home care could actually offer them in their situation as caregivers.

## Discussion

Based on the results, it could be assumed that the theoretical framework by Andershed and Ternestedt [15] involving the principal needs of family caregivers was successful for the design of the psycho-educational intervention. Participating in the intervention promoted caregivers’ knowledge about palliative care (knowing) and facilitated their situation in being close to a person with incurable illness both in relation to emotions (being) and managing and preparing for difficult situations (doing). According to Andershed and Ternestedt [15], when family caregivers “get to know” they develop insights which help in deciding how to be involved in the situation. It is reasonable to assume that such decisions are easier to make for family caregivers who feel more prepared. Preparedness was highlighted as something the intervention could promote by both professionals and family caregivers. Preparedness for caregiving in palliative care has been described as an important ongoing process through the entire illness trajectory and is intimately linked to preparedness for death and bereavement [15,29,30]. Family caregivers believed that the intervention could prepare them for the future deterioration of the patient. In order to prepare, caregivers are often in need of sensitive and appropriate communication including clear, reliable information, combined with relationship-centred care from health care professionals [31]. Andershed and Ternestedt [15] state that if family caregivers do not obtain information and do not “get to know” and to attain insight, their potential for supporting the patient in a way that is meaningful for both parties decreases. From the experiences of the participants, it would appear that the intervention could provide support for this process.

Health professionals stressed that working with the intervention demanded resources from them, in the form of time and engagement. They found it challenging to invite appropriate caregivers for the intervention. Based on this finding, it would seem as though the intervention had been experienced as being designed for a targeted group of family caregivers of patients approaching death. The intervention manual, based on the framework of knowing, being and doing, was developed for family caregivers living with a patient close to death, a situation to which not all family caregivers felt that they could relate in the same way. Recent developments in palliative care have recommended that this care philosophy is integrated earlier in the illness trajectory and for many different diagnoses. Rather than merely end-of-life care, palliative care is aimed at patients and their families at any age and at any stage of a serious illness, including those who are actively undergoing disease-targeted therapies [32]. However, the family caregivers’ experiences reveal that they found the intervention valuable even if the content was not always timely adjusted to their individual situation. With regard to this, the cautiousness demonstrated by health professionals to include family caregivers who were not in late palliative phase might have been unnecessary.

Participating in the delivery of the intervention was mainly described as a rewarding experience by both health professionals and family caregivers. Similar benefits of psycho-educational interventions for family caregivers have been confirmed in earlier studies [20,22]. However, the results from this study also show that intervention delivery had an emotional impact on health professionals, described as feelings of being lifted, confirmed and supported in their everyday work. The professionals also talked about gaining new insights from the intervention delivery. Insights like these could lead to increased opportunities for them to support families in palliative care, as has been formulated by Andershed and Ternestedt [15]. Another important aspect is that both health professionals and family caregivers, in various ways, described that the intervention led to closer relationships. These included relationships between family caregivers, but also between family caregivers and health professionals. Earlier research has shown that a trusting relationship between these two groups could promote a sense of security in family caregivers [10,33]. Organizing and delivering the intervention was also described as a way to focus on family caregivers within each setting and put their needs into the spotlight. Professionals in the study expressed a great commitment and enthusiasm in supporting family caregivers. When caregivers feel recognized and valued by health professionals with an approach characterized by respect, confirmation, openness and sincerity, Andershed and Ternestedt conclude that this may contribute to successful family caregiving [15].

The intervention’s structured design with the topics supported by the manual and the limited number of sessions was experienced both positively and negatively. The manual was described as a supportive tool and an effective framework for discussions, although health professionals did agree on the necessity to adjust it. Professionals stated that becoming familiar with the manual and preparing for the intervention sessions demanded a lot of time and engagement on their part and that they felt uncertain in the process of inviting family caregivers to the intervention. Based on this result, it seems like health professionals would have needed more comprehensive introduction to and training concerning the use of the manual, the intervention delivery and the inclusion criteria. This could be considered an important lesson to learn for future intervention designs in order to facilitate the contribution of health professionals. Using a manual could be considered an important part of a psycho-educational intervention because, in contrast to purely supportive interventions, it also has an educative component [34]. Manuals make it possible to make interventions more rigorous but it has also been stated that for successful interventions, it is important to take the group differences into account, something the health professionals all described that they had to do [9]. However, this could be seen as a limitation of the study, regarding fidelity to the intervention because it cannot be stated exactly how great the variations were in their delivery of the intervention. In the training of the professionals prior to delivering the intervention, it was stressed that all the topics of the manual should be covered. The personal qualities and the way professionals engaged family caregivers in the group processes could have had a potential influence on the participants’ experiences of the intervention. Because the intervention includes several components it could be considered complex and difficult to evaluate exactly what was the main influence of the participants’ experiences [23].

The credibility of this study could be affected by the fact that family caregivers were interviewed after they had participated in the intervention. In some cases the interviews took place several weeks after the intervention which could have influenced the caregivers’ recollection of the sessions. The credibility of focus group discussions with professionals involved in delivering the intervention could be influenced by the fact that the settings delivered the intervention a different number of times. Some of the professionals had only been involved in delivering one intervention. Method triangulation was applied in the study, with both individual interviews and focus groups used to collect data, which is considered something that could enhance the confirmability and dependability of the results [35]. The transferability of the study was strengthened through descriptions of the settings of the intervention, the number of participants in the study, the data collection methods and the time period when data was collected. The findings are presented in thorough descriptions with appropriate quotes, which has also been described as a way to enhance transferability [35]. The intervention was delivered in 10 different specialized palliative care settings, which strengthens the likelihood that the results should be transferable to other settings that specialize in palliative care. It is also likely that the results could be transferred to other settings where family caregivers are heavily involved, such as in general palliative care or dementia care.

## Conclusions

In conclusion, participating in the theoretically-based psycho-educational intervention in palliative home care was successful by means of being a positive and rewarding experience for both health professionals and family caregivers. Principally, the results reveal congruence between health professionals’ delivery and family caregivers’ participation in the intervention. The theoretical framework of Andershed and Ternestedt with the principal components, knowing, being and doing seems to be appropriate for designing interventions aiming to support family caregivers. From the perspective of family caregivers, the intervention provided knowledge in palliative care and practical and emotional support. Similarly, from the perspective of health professionals, the intervention had important positive effects on family caregivers and although the delivery demanded personal effort and engagement on their part, it also brought personal satisfaction and insights to them in their professional role.

The perspectives from this study provide important knowledge for future designs of psycho-educational interventions. The results indicate that interventions should be designed for both targeted and wider groups of family caregivers in palliative care, suitable to various diagnoses and different stages of the patient’s palliative trajectory, not just at the end of life. Using a manual gives structure to an intervention, but should be applied with regard to group differences and family caregiver needs. In order for health professionals to carry out interventions, they may be in need of support and supervision from their superiors. It is also necessary that professionals are given adequate time within their everyday clinical practice to deliver interventions as part of standard care aiming to support family caregivers.

## Abbreviation

FA: Framework analysis
